# A Case of Chagas Cardiomyopathy in Western Virginia: Worlds Away?

**DOI:** 10.7759/cureus.42158

**Published:** 2023-07-19

**Authors:** Kiriti Vattikonda, Christopher J Peterson, Benjamin Mulkey, Bradley Allen

**Affiliations:** 1 Internal Medicine, Virginia Tech Carilion School of Medicine, Roanoke, USA; 2 Cardiology, Virginia Tech Carilion School of Medicine, Roanoke, USA

**Keywords:** myocardial fibrosis, trypanosoma cruzi, heart block, chagas disease, chagas cardiomyopathy

## Abstract

Chagas cardiomyopathy, caused by the parasite Trypanosoma cruzi, is a significant cause of cardiac pathology worldwide. Though most frequently observed in Latin America, Chagas disease is present in the United States and should be considered in patients with heart block or other cardiac abnormalities and previous travel to or residence in endemic areas. Here we describe a new diagnosis of Chagas cardiomyopathy in a patient residing in Virginia with a previous residence in Mexico.

## Introduction

Chagas cardiomyopathy is a protozoal infection caused by Trypanosoma cruzi and is transmitted by the Triatomine insect. The clinical course of Chagas varies widely with the majority of patients remaining asymptomatic. In clinically significant disease, it can progress on a spectrum from less severe conduction defects and mild segmental wall motion abnormalities to more severe pathologies such as heart failure from dilated cardiomyopathy and fatal ventricular arrhythmias. Widespread fibrosis of the conduction system from Chagas can involve the sinoatrial (SA) and atrioventricular (AV) nodes and the bundle of His. The right bundle branch block is the most common intraventricular conduction abnormality [1]. In Latin America, it remains the most common cause of non-ischemic cardiomyopathy and a major cause of bradyarrhythmias requiring pacemaker implantation [2]. Over six million people are infected with Chagas disease worldwide (mostly in Central and South America), with greater than 300,000 affected in the United States [3]. Here we present a case of chronic Chagas cardiomyopathy occurring in a patient of Latin American ancestry without recent travel to his country of origin.

## Case presentation

A 62-year-old Hispanic male, originally from Mexico, presented with a six-month history of fatigue and dyspnea. He had no prior medical history and was not taking any prescription medications prior to admission. He denied any recent travel history or sick contacts. His physical exam was notable for bradycardia, no appreciable murmurs, and mild bilateral lower extremity edema. Notable labs included pro-BNP of 6341 pg/ml (reference range < 125 pg/ml) and negative troponin (< 0.30 ng/ml). The patient's initial ECG showed a normal sinus rhythm with a complete atrioventricular (AV) block and a prolonged QRS duration of 164 ms. Given ECG findings, cardiac electrophysiology was consulted for consideration of pacemaker implantation.

**Figure 1:**
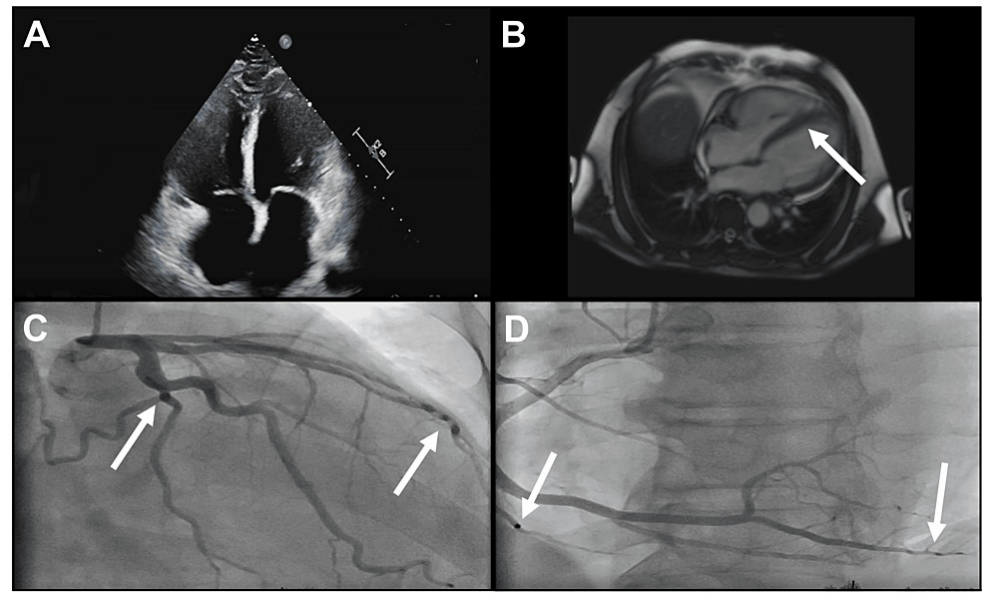
Cardiac imaging in a patient with Chagas cardiomyopathy (a) Transthoracic echocardiogram in four-chamber view showing dilation of all chambers. (b) Cardiac magnetic resonance imaging (MRI) showing myocardial fibrosis of the interventricular septum (c) and (d) coronary angiography showing non-occlusive anatomy in the arterial territory of the left main and its branches as well as the right coronary arteries.

Chest X-ray showed pulmonary edema and moderate cardiac enlargement. Transthoracic echocardiogram (TTE) showed dilated cardiomyopathy with severely reduced ejection fraction (EF) of 20%-25% (Figure 1A). A broad differential of etiologies was considered including Lyme carditis, sarcoidosis, lamin A/C (LMNA) cardiomyopathy, and giant cell myocarditis. Of note, the patient had last been in Mexico three years prior. Cardiac MRI revealed an area of mid-myocardial fibrosis limited to the basal interventricular septum (Figure 1B). Coronary angiography showed non-obstructive coronary artery disease (Figures 1C-1D). Lyme disease serologies returned negative. However, Trypanosoma cruzi antibody returned positive several days later suggesting trypanosomiasis. The patient underwent implantation of a cardiac resynchronization therapy-defibrillator (CRT-D) for definitive management of advanced conduction disease and primary prevention of sudden cardiac death, given the severely reduced EF. He was discharged home with losartan (25 mg, daily), metoprolol succinate (25 mg, daily), and furosemide (40 mg, daily) as part of guideline-directed medical therapy (GDMT) for heart failure with reduced ejection fraction (HFrEF). At three-month follow-up, the patient was free from heart failure symptoms and was cleared to return to work.

## Discussion

Chagas cardiomyopathy is largely thought of as a tropical disease process limited to the Central and South American regions. However, an estimated 300,000 individuals are infected with Trypanosoma cruzi in the United States, with 30,000-45,000 Chagas cardiomyopathy cases [4]. Chagas is also endemic to the United States and is transmitted by the Triatomine insect ('kissing bugs') [3]. While most often seen in the Southwestern United States, triatomine insects are also found in Eastern states such as Virginia [5]. Virginia was reported to have a notable rise in Chagas cases in the early 2010s, with an estimated 7,300 cases in 2012 [6], in part due to an immigrant population with suspected previous exposure [7]. It is important to recognize it as a major etiology of non-ischemic cardiomyopathy with or without advanced conduction system disease, especially in patients who were born or spent significant time in endemic regions. In non-endemic areas, means of transmission include blood transfusion, organ transplantation, and vertical transmission [8].

Diagnosis depends on the phase of disease pathophysiology. In the acute phase, which can last 40-60 days, microscopic identification of Trypanosoma cruzi is considered the gold standard [8]. Concentration methods, such as microhematocrit, and PCR are also diagnostic options [9]. In the chronic (or indeterminate) phase of Chagas, antibody testing via methods such as enzyme-linked immunosorbent assay (ELISA) is used due to the presence of trypanosomes in organ tissues or absence from the host [9]. Confirmation with at least two assays is recommended [9]. In this case, the patient's clinical picture, previous residence in an endemic area, and positive Trypanosomacruzi serology were considered sufficient by our team for the diagnosis of Chagas cardiomyopathy. In newborns with serologically positive mothers, PCR is preferred (due to transplacental transfer of IgG) or serology at nine months [10]. Progression to cardiomyopathy in patients with chronic Chagas occurs in up to 40% of patients at a rate of 2% to 7% per year [3,10]. Given this, some experts recommend surveillance with ECG and chest X-ray every few years to monitor for progression to Chagas cardiomyopathy [10]. There is no Chagas cardiomyopathy-specific biomarker [10,11]. At the time of diagnosis of Chagas disease, most experts recommend a combination of ECG, echocardiography (especially transesophageal), and 24-hour Holter monitoring [10-12]. In asymptomatic patients, abnormal ECG is often the first indication of Chagas cardiomyopathy [8]. The presence of a right bundle branch block or a left anterior fascicular is more frequently observed in arrhythmias in Chagas cardiomyopathy [13].

Treatment for Chagas cardiomyopathy mostly focuses on the treatment of cardiac pathology. While the anti-parasitic drugs benznidazole and nifurtimox are effective against Trypanosoma cruzi, they are largely ineffective at improving cardiovascular pathology by the time Chagas has progressed to cardiomyopathy, a process which typically takes several years. In a prospective, multicenter, randomized trial with 2,854 Chagas cardiomyopathy patients (BENEFIT trial), treatment with benzimidazole did not significantly reduce hospitalizations, major adverse cardiovascular events, and cardiovascular mortality compared to placebo [14]. Patients with Chagas cardiomyopathy should be managed according to their respective cardiac pathology [3]. However, some Chagas cardiomyopathy-specific trials have been performed. For example, beta-blocker therapy has been shown to improve survival [15,16]. There is limited information on antiarrhythmic therapy for Chagas cardiomyopathy, with amiodarone being the most frequently studied therapy [11]. In patients with Chagas cardiomyopathy, conduction deficits are increasingly common, and implantable cardioverter-defibrillators (ICDs) are effective in preventing secondary fatal arrhythmia [17]. The number of defibrillator shocks in 30 days is a predictor of mortality [18]. Finally, while Chagas cardiomyopathy patients are at a higher risk for thromboembolism (up to 2.7% per year) [10], anticoagulation is not usually recommended unless the patient develops atrial fibrillation, intracardiac thrombus, apical aneurysm, systolic dysfunction, or history of stroke [3]. Saraiva et al. created a scoring system to evaluate for primary stroke prophylaxis based on left ventricular (LV) systolic dysfunction, apical aneurysm, primary ST changes, and age > 48 years [19].

Several scoring systems have been developed to predict mortality in Chagas cardiomyopathy [10], with the six-category Rassi score [20] arguably being the most notable (Table 1). This patient was considered intermediate risk (7 points) given male gender and cardiomegaly on chest X-ray. Other staging systems for cardiomyopathy include the Brazilian Expert Consensus, the Latin American Guideline, Kuschnir, and the modified Los Andes classification [10]. All of these classifications use a combination of ECG, echocardiogram, heart size on chest X-ray, and/or heart failure symptoms. Of note, patients with Chagas cardiomyopathy may have a worse prognosis than non-Chagas dilated cardiomyopathy patients, possibly due to the extent of cardiac remodeling including cardiac cell lysis, intracardiac neurological damage, and cardiac microcirculatory lesions [11].

**Table 1:** Rassi score for Chagas cardiomyopathy [20] Risk stratification- low risk (0 to 6 points), intermediate risk (7 to 11 points), and high risk (12 to 20 points) VT: ventricular tachycardia; WMA: wall motion abnormality; NYHA: New York Heart Association

Feature	Points
Male sex	2
Low QRS voltage	2
Nonsustained VT	3
Segmental or global WMA	3
Cardiomegaly	5
NYHA class III or IV	5

## Conclusions

This clinical case highlights the importance of considering Chagas disease in the differential of non-ischemic cardiomyopathy and advanced conduction system disease in Central and South American patients. Though less common in the United States, the nevertheless endemic nature of the disease and increasing prevalence of international migration makes this pathology, though seemingly 'worlds away', a legitimate concern for cardiac dysfunction. Further clinical trials and research are required to develop more targeted diagnostic and treatment options to help effectively alter the progression of this disease.
